# Doping Effects of Carbon Nanotubes and Graphene on the Flexural Properties and Tribological Performance of Needle-Punched Carbon/Carbon Composites Prepared by Liquid-Phase Impregnation

**DOI:** 10.3390/nano13192686

**Published:** 2023-09-30

**Authors:** Kuo-Jung Lee, Mu-Chou Lee, Yung-Hui Shih, Hsun-Yu Lin

**Affiliations:** Department of Materials Science and Engineering, I-SHOU University, Kaohsiung 84001, Taiwan; a5013486@gmail.com (M.-C.L.); yhshi@isu.edu.tw (Y.-H.S.); carbonfish028@gmail.com (H.-Y.L.)

**Keywords:** carbon nanotubes, graphene, tribological performance, C/C composites

## Abstract

The main goal of this study is to investigate the doping effects of carbon nanotubes (CNTs) and graphene on the needle-punched carbon/carbon (C/C) composites that are prepared by liquid-phase impregnation. In order to achieve, for the C/C composites, the purposes of high flexural strength, stable friction coefficient, low weight loss, and high thermal conductivity, our primary concern is to examine the flexural properties and the tribological performance, and then to explore a little further into the influence on thermal conductivity. In this study, carbon fiber preforms were first fabricated by needle-punched carbon-fiber cloth, and then liquid-phase phenolic resin, doped with different proportions of carbon nanotubes and graphene, was used as the impregnation solution to carry out multiple densification (impregnation–carbonization) cycles and fabricate various C/C composites. The main purpose was to probe into the doping effects of the CNTs and graphene, added to the impregnation solution, on the properties of C/C composites. The experimental results show that the addition of CNTs and graphene can improve the heat conductivity, flexural properties, and tribological performance of C/C composites, and the impact on these properties is more significant with the addition. Furthermore, the properties of graphene-doped C/C specimens are better than those of CNT-doped C/C specimens.

## 1. Introduction

Carbon/carbon (carbon fiber-reinforced carbon matrix, C/C) composites with excellent thermal, mechanical, and tribological properties fabricated through liquid carbon-precursor impregnation or chemical-vapor infiltration (CVI) are currently the best choice of materials in aircraft brake disks [1,2,3].

Research on the tribology of carbon/carbon composites can be generally divided into the following three categories: (1) effects of process parameters (such as different additives [4,5,6,7,8,9], manufacturing processes [10,11,12,13,14,15], processing temperatures [16,17,18,19], and carbon fiber types [20,21], etc.), wear parameters [17,22,23,24,25,26,27], and environmental factors [28,29,30,31] on the tribological behavior; (2) observation of the microstructure of worn surfaces and debris [32,33,34,35,36,37,38,39]; and (3) investigation of the basic friction, wear behavior, and wear mechanism [7,40,41,42]. The author’s earlier studies [13,14,15] were to investigate the effects of densification parameters such as the impregnating precursors, impregnation method, densification cycles, carbonization temperature, and rapid carbonization on the tribological behaviors of C/C composites.

The CNTs and graphene, allotropes of carbons with excellent chemical, physical, mechanical, and tribological [43,44] properties, have been widely reported to improve the wear resistance and strength of composites when they are mixed in polymer [45,46,47,48], metal [49,50,51], and ceramic [52,53,54] materials. However, very limited discussion, based on our understanding, has been made about the correlation between the mechanical properties, especially tribological behaviors, and the addition of CNTs or graphene in the C/C composites. Zhang et al. [55,56] observed that the addition of suitable CNT content improved the interface with CVI pyrolytic carbon and enhanced the mechanical properties of C/C composites. Lim et al. [4] reported that in the CNT/carbon composites fabricated by infiltration of slurry mixed with multi-walled CNT and phenolic resin, the wear loss decreased significantly, but the friction coefficient slightly increased as the addition of CNT was augmented. Gong et al. [7,9] reported that in the CNT-doped C/C composites fabricated by chemical-vapor infiltration (CVI), the CNTs can not only increase wear resistance but also maintain stable friction coefficients under different loads. Furthermore, there is a lack of literature on the research topic of adding graphene to C/C composites because few attempts have so far been made to this point. Singh et al. [57] have discussed the effect of graphene size on the formation of C/C composites. The results show that graphene size will affect the graphitizing/non-graphitizing structures in the matrices of C/C composites.

The main purpose of this study is to fabricate the CNTs-doped and graphene-doped C/C composites prepared through the needle-punched method, liquid-phase infiltration, and fast carbonization process. It is our objective in this experiment to research the interrelationships among the tribological properties, mechanical properties, microstructures, and process parameters of self-made C/C specimens.

## 2. Materials and Methods

### 2.1. Specimen Preparation

#### 2.1.1. Preparation of Impregnation Solution

Regarding the dispersion method of nanocarbon materials in polymers, an effective dispersion method was proposed in the literature [58]. However, in this study, the impregnation solution containing nanocarbon materials needs to infiltrate the carbon fiber preform subsequently. Therefore, it is advisable not to add an excessive amount of nanocarbon filler to prevent poor dispersion of the nanocarbon filler and to avoid the impregnation solution becoming too viscous, which could lead to difficulties in the subsequent impregnation process. In this experiment, the liquid-phase resole-type phenolic resin (Texxco. Enterprise Co., RM-18389, New Taipei City, Taiwan) was used as the solution of carbon-precursor impregnation and then applied, respectively, to the multi-walled CNTs (Golden Innovation Business Co., C tube-015, New Taipei City, Taiwan) and the multi-layered graphene (Enerage Inc., P-ML20, Yilan, Taiwan) with different doping proportions (0, 1, 5, 9 wt%). During the process, ethyl acetate (EA) (TEDIA Company, EH3857-150, Fairfield, OH, USA) was added as a co-solvent, and a mixer (Eyela, Mazela Z., Tokyo, Japan) was used for stirring at a speed of 400 rpm for 10 min, so that the CNTs and graphene were uniformly dispersed in the liquid phenolic resin.

#### 2.1.2. Carbon Fiber Preform Preparation

To begin with, the needle-punched integrated carbon fiber cloth was used as carbon fiber skeletons, which were fabricated by alternatively stacking eight layers of carbon fiber cloth (Mitsubishi Rayon, TR 30S 3L, Tokyo, Japan) and seven layers of short-cut fiber (Toray, T700S, Tokyo, Japan) by using the needle-punched technique. In this study, the carbon fiber types were all PAN-based carbon fiber. Then, the carbon fiber skeletons were impregnated with liquid-phase phenolic resin mixed with different proportions of carbon nanotubes and graphene. The impregnated carbon fiber skeletons were further press-molded at 180 °C under a unidirectional pressure of 100 MPa to make round disks of 25.4 mm in diameter and of desirable thickness. These as-cured specimens were then carbonized in the furnace with a heating rate of 3 °C/min to a temperature of 1000 °C and held at this temperature for 120 min under a nitrogen atmosphere to make porous preforms.

#### 2.1.3. Densification

Porous preforms were further densified by the vacuum/pressurized impregnation process. During the vacuum impregnation process, the porous preforms were placed in a stainless steel vacuum chamber. The chamber was evacuated and then filled with impregnation solution for the vacuum impregnation. Then, the preforms after the vacuum impregnation were further placed in a pressurized impregnation mold containing impregnation solution, and the pressure was maintained at 100 MPa for 30 min so that the impregnation solution further infiltrated into the pores inside the specimen by the pressurized impregnation. Each impregnated specimen was cured and dried before carrying out the fast carbonization. The fast carbonization described in the author’s earlier literature [15,59] was followed by heating the impregnated specimens in a nitrogen atmosphere to 1000 °C at a heating rate of 1000 °C/min and holding the temperature for 30 min. The densification (impregnation-carbonization) cycles of each individual specimen were repeated up to four times. After each cycle was completed, it was necessary to remove the fine impurity carbon layer on the surface of the specimen by grinding and ultrasonic vibration cleaning to prevent the impurity carbon layer from blocking the pores and forming closed pores in the specimen. In the following study, the specimens prepared from the liquid-phase resole-type phenolic Resin doped with different proportions (0, 1, 5, 9 wt %) of CNTs and graphene were abbreviated and designated, respectively, as the R, RC1, RC5, RC9, RG1, RG5, and RG9 specimens. The capital letter R indicates the specimen without doping of CNTs or graphene, and the RC and RG denote, respectively, the CNTs-doped and graphene-doped specimens.

### 2.2. Characterization

#### 2.2.1. Density and Porosity Measurement

The density and open porosity of each specimen were measured by the water immersion method according to ASTM C-20 [60].

#### 2.2.2. Measurement of Thermal Conductivity

The thermal conductivity of each specimen was measured using a thermal conductivity analyzer (Hot disk, TPS 2500S, Göteborg, Sweden) with the function of a transient-plane heat source (ISO 22007-2 [61]). Prior to the measurement, all specimens were mechanically polished through grit papers to make their surfaces flat. A Kapton-type sensor, placed between two pieces of specimens, was used, and then an input power of 50 mW was applied for 2–4 s to measure the thermal conductivity.

#### 2.2.3. Flexural Strength Tests

The flexural strength was determined by using the three-point bending test with a load speed of 4 × 10^−2^ mm/s, according to the specification of ASTM C1341-06 [62] on the Instron test machine (Shimadzu, AGS-500A, Kyoto, Japan). The size of the specimen bar is 26 × 3 × 1 mm^3^, and the length of the support span is 16 mm. The flexural strength of specimens (S_U_) is calculated in accordance with the equation listed in the following:

S_U_ = 3P_U_L/(2bd^2^)

where P_U_ is the maximum load, L is the length of the support span, and b and d are the width and thickness of the specimens, respectively.

#### 2.2.4. Friction and Wear Tests

The friction and wear tests were operated on a homemade disc-on-disc sliding wear tester. A schematic diagram and specifications were described in the author’s earlier literature [14]. This wear tester consists of two parts: the rotor shaft, which is connected to a motor to control the rotation speed, and the stator shaft, which is connected to a pneumatic system to control the applied load. During the wear test, two specimens with the same processing conditions are fixed to the rotor shaft and stator shaft to rub against each other. Multiple continuous wear tests were conducted under the same wear condition in this study. A fixed load of 0.8 MPa, a constant rotor speed of 600 rpm (linear speed is 0.4 m/s), and a testing time of 300 s (sliding distance is 120 m) were used in every wear test. Each specimen was subjected to wear tests 15 times.

Prior to the first testing, all specimens were mechanically polished through a level of #1200 grit paper, followed by ultrasonic cleaning and drying. A strain gage equipped with an LRK-100K load cell (NTS Technology, Nara, Japan) was used to determine the friction coefficient. The friction coefficient μ can be reduced to the formula:

μ = M/F_n_r

where M is the torsional moment, F_n_ is the load, and r is the radius of the specimen. After each wear test, the area under the curve of friction coefficient was first calculated by means of integration and divided by the test time, namely, the average friction coefficient for this test. Next, we calculated the values of 15 wear tests, respectively, and took their mean value as the total average friction coefficient of the specimen. Since the rotor and the stator were always of identical material, the weight loss from both discs was measured and averaged for each wear test.

#### 2.2.5. Microstructure

A scanning electron microscope (SEM) (Hitachi, FE-SEM4700, Tokyo, Japan) with a cold-field emission emitter was used to observe the fracture surface morphologies of different specimens after the bending test. A SEM (Hitachi, S-3400, Tokyo, Japan) was also applied to examine the morphologies and worn surfaces of different specimens after experiencing multiple continuous wear tests.

## 3. Results and Discussion

### 3.1. Porosity and Density

**Figure 1:**
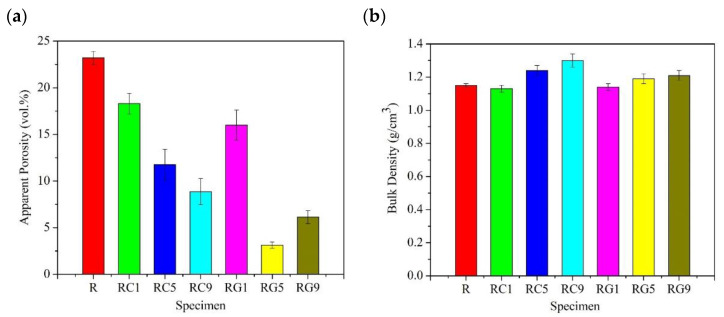
The (**a**) porosity and (**b**) density of specimens.

As shown in Figure 1a,b, we observe that the R specimen fabricated only with pure phenolic resin as the impregnation solution has the highest apparent porosity and the lowest density. Compared with the R specimen, both series of RC(RC1, RC5, RC9) and RG(RG1, RG5, RG9) specimens, added with CNTs and graphene in the impregnation solution, show a trend of decreasing apparent porosity and increasing density; moreover, this tendency is more significant when their addition proportions are larger. It shows that the impregnation solution, with CNTs or graphene, effectively fills the pores of specimens by the vacuum/pressure impregnation process and ameliorates the densification of specimens.

Further investigation of the RC- and RG-series specimens reveals that the apparent porosity of RG-series specimens is slightly lower than that of RC-series specimens. It helps account for the result that graphene has a large area of flake structure to cover and fill, effectively, the pores inside specimens when it is infiltrated into the specimens.

### 3.2. Thermal Conductivity

**Figure 2:**
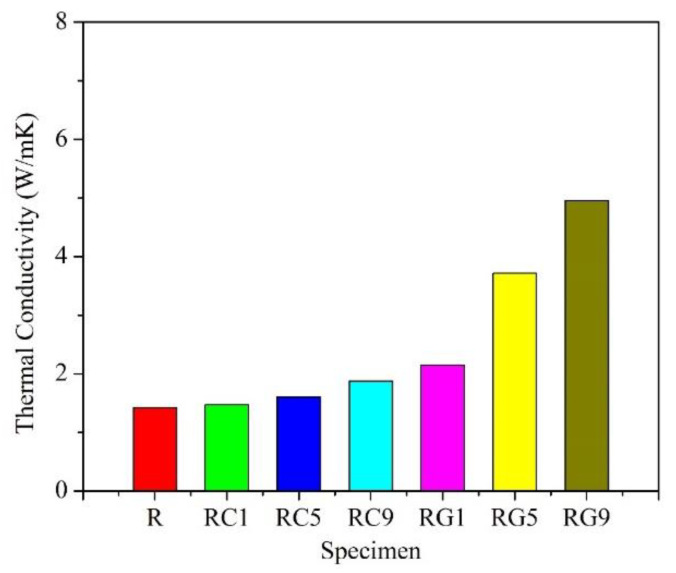
The thermal conductivity of specimens.

Figure 2 shows the thermal conductivity of each specimen. Among all of the specimens, the thermal conductivity of the R specimen is the lowest (1.4 W/mK), and it is reasonable to explain that the pores of the specimen become obstacles to heat transfer due to the high porosity of the R specimen. The thermal conductivities of RG- and RC-series specimens increase with the addition of the second reinforcement; moreover, the RG-series specimens have higher values than the RC-series ones. The most likely explanation is that the thermal conductivity of the multilayered graphene (5300 W/mK) [63], added in RG series, is larger than that of the multi-walled CNTs (3000 W/mK) [64,65], added in RC series. In addition, there is further evidence to suggest that the porosity of RG-series specimens is also lower than that of RC-series specimens, which are all the key factors beneficial to the better thermal conductivities of the RG-series specimens.

### 3.3. Flexural Strength

**Figure 3:**
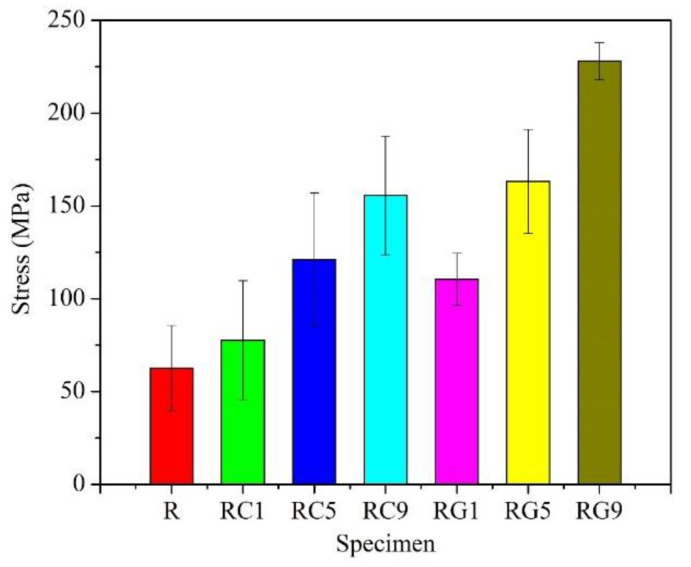
The flexural strength of specimens.

**Figure 4:**
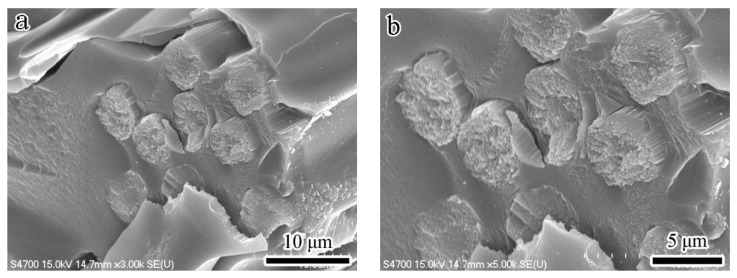

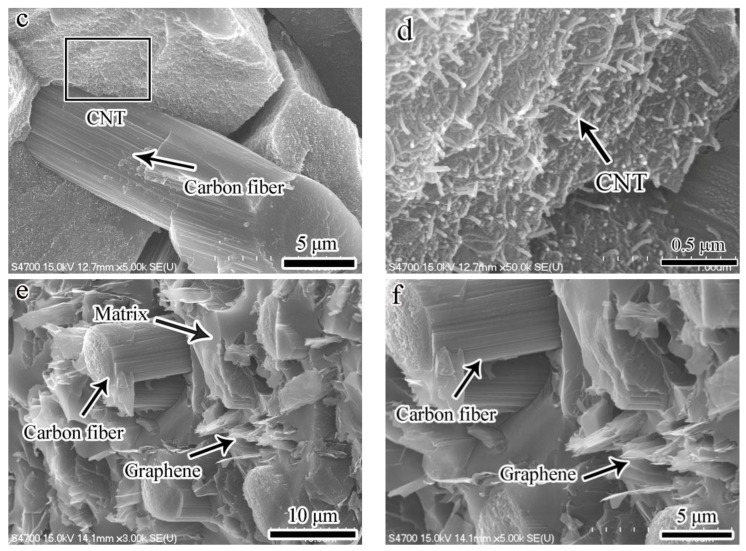
Fracture surface morphologies of specimens (**a**,**b**) R specimen; (**c**,**d**) RC-series specimens; (**e**,**f**) RG-series specimens.

As shown in Figure 3, the R specimen demonstrates the lowest flexural strength (about 62 MPa) of all the specimens. The morphology of the fracture surface for the R specimen after the flexural test is quite flat (Figure 4a,b), and it exhibits the phenomenon of brittle fracture. The flexural strength of RC- and RG-series specimens also increases with the addition of the second reinforcement. These experimental results illustrate a key point: the doping of CNTs or graphene improves, assuredly, the flexural strength of the specimens.

Further comparing the specimens with the same content of reinforcement, the RG-series specimens still show higher values of flexural strength than the RC-series specimens. The best account for this result can be clarified in the following three aspects: First, the porosity of RG-series specimens (about 3~16%) is lower than that of RC-series ones (about 8~18%), which shows that the structure of RG-series specimens is denser and its flexural resistance is better. Secondly, it is easier to be pulled out of the matrix in the flexural tests of RC-series specimens due to the small size of CNTs (about 1–10 μm in length), and the flexural strength of RC-series specimens is therefore lower. Relatively, as described in the literature [48], graphene has a larger surface area than carbon nanotubes and is more easily modified by different molecules. In this study, the size of the raw flake graphene used ranged from 9–40 μm, resulting in a larger contact area and greater adhesion force with the matrix compared to carbon nanotubes. Therefore, more additional force is required to draw out the graphene during a flexural test, and it helps to significantly enhance the flexural strength of RG-series specimens. Thirdly, according to the microstructure observation of the fracture surface after the flexural test, it is clear that the multi-walled CNTs are mostly distributed in the matrix (Figure 4c,d). Although they can improve the flexural strength of the specimen, their effect is limited. However, the multi-layered graphene is embedded in the interface between the carbon fiber and the matrix (Figure 4e,f), which plays an auxiliary role in strengthening the interface interaction. Therefore, more force is required to break the RG-series specimens during the flexural test, and the flexural strength of the RG-series specimens is also improved.

### 3.4. Friction and Wear

**Figure 5:**
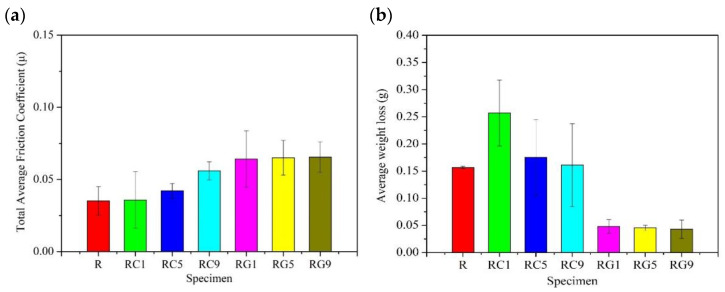
The (**a**) total average friction coefficient and (**b**) wear data of specimens during multiple continuous wear tests.

Figure 5 shows the average friction coefficient and weight loss of each specimen under multiple continuous wear tests, respectively. The total average friction coefficient of the R specimen is about 0.035, and the weight loss is nearly 0.16 g, while the total average friction coefficients of the RC- and RG-series specimens are both higher than those of the R specimen. The friction coefficients of RC-series specimens exhibit an upward trend with the addition of CNTs, while the weight losses show a downward tendency. This phenomenon is accounted for by the fact that the weight losses of specimens are reduced if the wear debris containing CNTs and carbon matrix is repeatedly rolled and compacted to produce lubricative films during the wear process. However, the hardness of wear debris containing CNTs is higher. Before the wear debris forms a stable lubricative film during the wear process, the harder wear debris still damages the surface and increases the weight losses of the specimens; therefore, the overall weight losses of the RC-series specimens are still higher than those of the R specimen. It is worth noting that the standard deviation of the average weight losses of the RC-series specimens in Figure 5b is very large. It reveals that weight losses of the RC series change greatly during each wear test, which also implies that more wear debris containing CNTs is needed to form the lubricative films in the RC-series specimens, and the lubricative films are also relatively unstable.

Compared with the R and RC-series specimens, the weight losses of the RG-series specimens lessen significantly. The possible reason is that the wear debris containing flake graphene makes it easier to form a dense lubricative film during the wear process. This lubricative film helps stabilize the wear behavior and further reduce the weight losses of the specimens. Furthermore, it is apparent that in the RG-series specimens, the total average friction coefficients, or weight losses, of the three specimens are mostly similar. It indicates that even adding a small amount of graphene helps the specimen form a lubricative film during the wear process. This film has an excellent lubricating effect, which can not only reduce weight loss but also further stabilize the friction coefficient.

### 3.5. Surface Morphology

**Figure 6:**
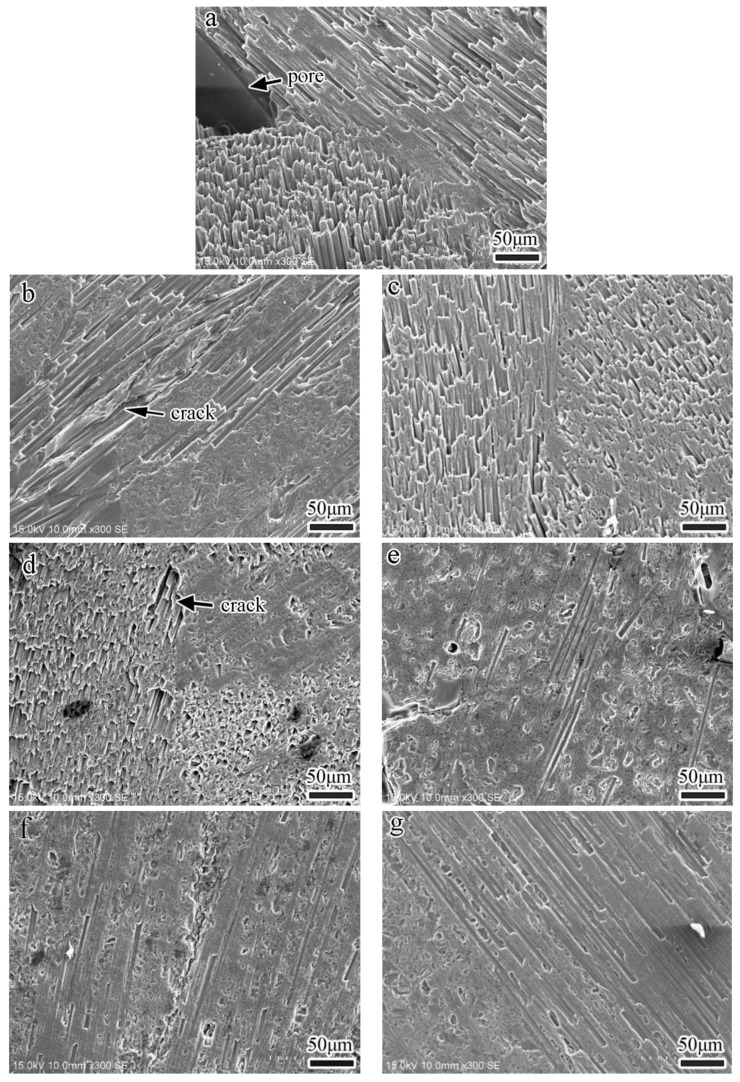
Surface morphologies of specimens before multiple continuous wear tests. (**a**) R specimen, (**b**) RC1 specimen, (**c**) RC5 specimen, (**d**) RC9 specimen, (**e**) RG1 specimen, (**f**) RG5 specimen, (**g**) RG9 specimen.

Figure 6 shows the microstructure of the various specimens before the wear test. It is evident that the surfaces of various specimens after four densification processes mostly exhibit dense and smooth morphologies, but some small cracks/pores within the matrix and between individual fibers are still visible. As indicated by the arrow in Figure 6a, larger pores or cracks resulting from the decomposition and thermal shrinkage of the resin matrix after carbonization are observed in the R specimen. It also corresponds to the fact that the porosity of the R specimen is the highest among all specimens. The pores/cracks of RC- and RG-series specimens are apparently less than those of the R specimen, especially since the surfaces of RG5 and RG9 specimens display denser and smoother morphologies (Figure 6d,e), which also echo the outcome of less porosity in these two series of specimens in Figure 1a.

**Figure 7:**
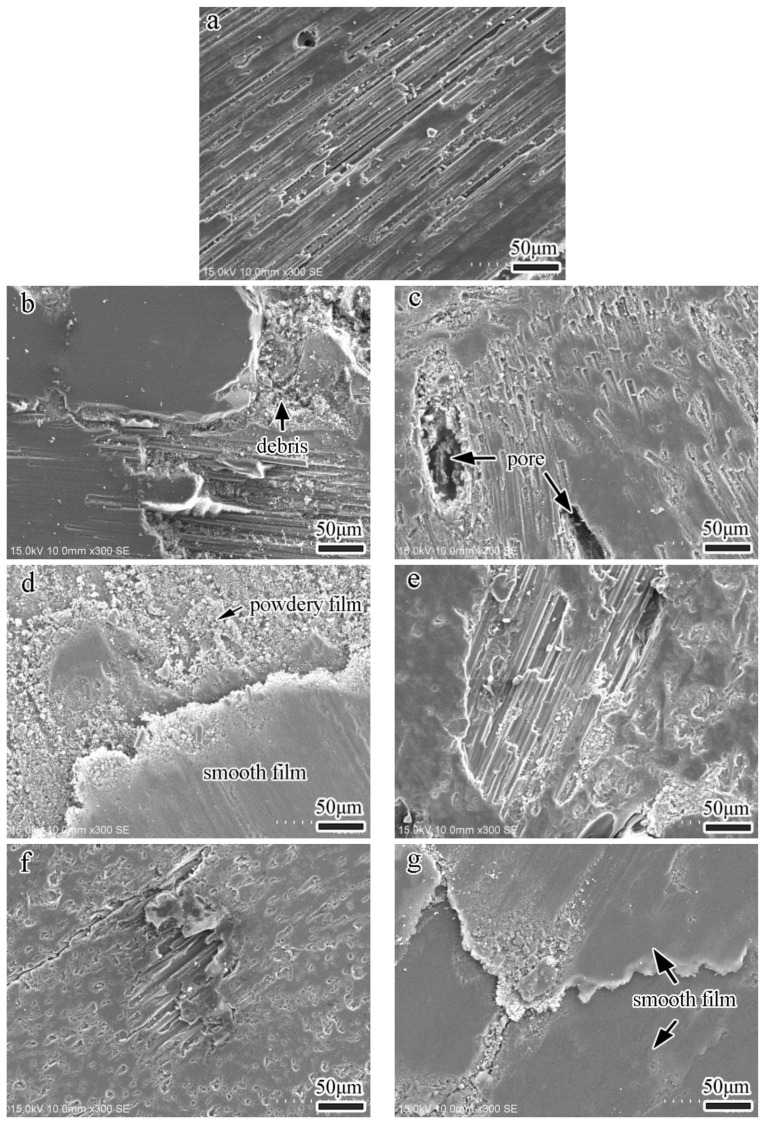
Worn surface morphologies of specimens after multiple continuous wear tests. (**a**) R specimen, (**b**) RC1 specimen, (**c**) RC5 specimen, (**d**) RC9 specimen, (**e**) RG1 specimen, (**f**) RG5 specimen, (**g**) RG9 specimen.

Figure 7 shows the worn surface morphologies of various specimens after experiencing multiple continuous wear tests. As shown in Figure 7a, only a little wear debris is generated on the worn surface of the R specimen. The fibers on the worn surface are less than those before the wear test, and the worn surface appears to be flattened. This type of worn surface was responsible for the low average friction coefficient and less weight loss, as indicated in Figure 5, during the wear process.

Figure 7b–d demonstrates the microstructures of RC-series specimens after a wear test. It is apparent that the worn surfaces of RC1 and RC5 specimens are relatively chaotic. Although there is a large area of lubricative film, there is also more wear debris, pores, and cracks on the worn surfaces (Figure 7b,c). With the addition of CNTs, a thicker lubricative film form ons the worn surface. It is evident that in the RC9 specimen (Figure 7d), the wear debris is piled up, rolled, and compacted to form two types of smooth and powdery lubricative films, covering the surface of the specimen. Hence, the fibers are no longer clearly visible. According to an earlier study [29], the formation of the smooth lubricative film helps to reduce the weight loss of the specimen, while the powdery lubricative films are accompanied by a relatively high friction coefficient. This explains why the weight loss of the RC9 specimen is lower than that of the RC1 and RC5 specimens, but the friction coefficient is higher.

Figure 7e–g displays the microstructures of the worn surfaces of RG-series specimens. It is obvious that the worn surfaces of the RG-series specimens are smoother than the R and RC-series specimens, and there is less wear debris on the surfaces. The surfaces of the RG1 and RG5 specimens are covered with thin lubricative films, and only a few pieces of wear debris are stuck between the carbon fibers; however, the worn surface of the RG9 specimen (Figure 7g) is covered with a thicker and smoother lubricative film. The relatively stable worn surfaces of the RG-series specimens are also responsible for the similar friction coefficients and lower weight losses of this series of specimens in Figure 5.

## 4. Conclusions

The CNTs-doped (RC) and graphene-doped (RG) carbon/carbon specimens in this study were fabricated by employing needle punching and liquid-phase impregnation. The results show that the specimens in both series, added, respectively, with CNTs or graphene in the impregnation solution, exhibit a trend of decreasing apparent porosity and increasing density. The thermal conductivity and flexural strength of CNTs- and graphene-doped C/C specimens are both higher than those of the original (R) C/C specimen, prepared only from the pure liquid-phase resole-type phenolic resin. The thermal conductivity and flexural strength of both CNT- and graphene-doped C/C specimens increase with the addition proportions of the second reinforcement. Furthermore, the graphene-doped C/C specimens display better performances on thermal conductivity and flexural strength.

In summary, it is clear that the graphene-doped C/C specimens reveal more stable friction coefficients and lower weight loss than those of the other specimens during the multiple continuous wear tests. The worn surfaces of CNT-doped C/C specimens are rougher and more disordered than those of the other specimens; hence, they are accompanied by relatively higher weight losses. It follows from what has been said that a large area of thick and smooth lubricative films is easily presented on the worn surfaces of the graphene-doped C/C specimens, which is also responsible for their stable friction coefficients and lower weight losses. Based on these grounds, this experiment has accomplished, by doping the carbon nanotubes or graphene in C/C composites, a possible procedure to better the diverse properties, including, as mentioned previously, the larger flexural strength, stable friction coefficient, lower weight loss, and higher thermal conductivity.

## Data Availability

The data presented in this study are available on request from the corresponding author.
